# Do We Have Any Environmental or Perinatal Factor Which May Predispose for Paediatric Airways Diseases? An Italian Population Prospective Study

**DOI:** 10.1111/coa.70032

**Published:** 2025-09-14

**Authors:** Cecilia Rosso, Federica Turati, Alberto Maria Saibene, Elvira Verduci, Giuseppe Banderali, Monica Ferraroni, Giovanni Felisati, Carlotta Pipolo

**Affiliations:** ^1^ Department of Otorhinolaryngology Santi Paolo e Carlo Hospital, Università Degli Studi di Milano Milan Italy; ^2^ Pediatric Clinic, Department of Clinical Sciences and Community Health Università Degli Studi di Milano, Milan, Italy; Fondazione IRCCS Ospedale Maggiore Policlinico Milan Italy; ^3^ Department of Otorhinolaryngology, Santi Paolo e Carlo Hospital, Department of Health Sciences Università Degli Studi di Milano Milan Italy; ^4^ Pediatric Department, ASST Fatebenefratelli Sacco Università degli Studi di Milano Milan Italy; ^5^ Pediatric Department, Santi Paolo e Carlo Hospital Università Degli Studi di Milano Milan Italy

**Keywords:** acute otitis media, allergy, environment, paediatric ENT diseases, risk factors, upper respiratory tract infections

## Abstract

**Background:**

Paediatric airway diseases such as asthma, allergies, rhinitis, upper respiratory tract infections and acute otitis media are major health challenges for children globally. The prevalence of these conditions has been increasing, impacting children's quality of life, educational attainment and imposing a substantial economic burden.

**Objectives:**

This longitudinal prospective study investigated the prevalence rates and environmental links associated with paediatric airway diseases in the first 3 years of life in 241 newborns, with the goal of contributing to early detection, prevention and management strategies.

**Methods:**

Structured questionnaires were administered to parents at birth, 1 year and 3 years of age. Data on socioeconomic factors, pregnancy and delivery characteristics, parental smoking, breastfeeding, childcare attendance and children's health history were collected. Skin prick tests were conducted in year 3 to assess allergic sensitisation.

**Results:**

Two hundred seven patients completed three‐year follow‐up. Factors such as having siblings, exclusive breastfeeding and attending kindergarten were associated with increased risks of certain diseases at 1 and 3 years. Smoking exposure appeared protective against wheezing in the first year. Breastfeeding showed mixed results, with protective effects against URTIs at 1 year but a potential risk factor for asthma at 1 year. Kindergarten attendance was associated with increased risks of URTIs and AOM at 3 years but appeared protective against inhalant allergies.

**Conclusion:**

The study highlighted the complex interplay of various factors in the development of paediatric airway diseases. Further research is needed to refine our understanding of these factors and their impact on paediatric diseases.

**Summary**

- *Paediatric airway diseases*: Conditions such as asthma, rhinitis, URTI and AOM are increasingly prevalent among children, with air pollution and global warming contributing significantly to their exacerbation.
- *Longitudinal study*: The study followed 241 newborns for 3 years, examining the influence of factors like breastfeeding, smoking exposure, siblings and daycare attendance on the development of paediatric airway diseases.
- *Smoking exposure*: While prenatal and postnatal smoking exposure increased the risk of URTIs, surprisingly, it was inversely associated with asthma/wheezing in the first year of life, highlighting possible age‐related differences.
- *Daycare and sibling impact*: Attending daycare raised the risk of AOM and bronchitis but reduced the likelihood of allergies to inhalants by age three, while having siblings increased the risk of asthma and bronchitis in early life.
- *Breastfeeding's Dual Role*: Exclusive breastfeeding was protective against URTIs but was linked with a higher rate of asthma/wheezing in the first year, showing conflicting effects based on the condition analyzed.

## 1 Introduction

Paediatric airway diseases such as asthma, allergies, rhinitis, upper respiratory tract infections (URTI) and acute otitis media (AOM) pose significant challenges to the health and well‐being of children worldwide. Allergic conditions like rhinitis and allergies to environmental triggers have also become more prevalent, rising to 20% in schoolchildren and 35% in adolescents [1].

These conditions are part of a broader clinical continuum known as the ‘global airway concept’, which recognises the functional and immunological interconnection between the upper and lower respiratory tracts. According to this model, inflammation in one segment of the airway can influence the other, supporting the idea of a unified respiratory mucosa with shared pathophysiological mechanisms.

The onset of airway diseases in childhood often reflects an imbalance in the early immune response, frequently skewed toward a Th2 phenotype in genetically predisposed individuals. This is further amplified by environmental triggers – such as air pollution, viral infections, allergen exposure and impaired epithelial barrier integrity – leading to chronic inflammation, sensitisation and tissue remodelling. If left untreated or inadequately managed, these inflammatory processes can progress in severity over time, resulting in the acquisition of comorbidities such as persistent asthma, allergic rhinitis, recurrent infections and even long‐term pulmonary function impairment [2].

Moreover, in recent years, increasing urbanisation and climate change have further contributed to the disruption of the epithelial barrier and the persistence of airway inflammation, particularly in vulnerable paediatric populations [3].

Therefore, understanding the early‐life factors that influence the onset and trajectory of these diseases is crucial [4]. Such factors could include genetic predispositions, early‐life environmental exposures, family history and lifestyle choices. Understanding these predictive factors can guide healthcare providers, policymakers and parents in implementing preventive measures and targeted interventions to mitigate the burden of paediatric airway diseases.

The EUFOREA consensus strongly advocates for shifting the focus of asthma care toward prevention and remission, aligning with the present study's goal of early identification of modifiable risk factors in childhood [5].

Literature already contains evidence about many risk factors that may affect the onset of certain pathologies such as asthma, but mostly retrospectively and focusing only on one issue [6]. There remains a significant gap in prospective, comprehensive research assessing multiple environmental and familial influences across a range of respiratory diseases during the critical first years of life.

In this article, we longitudinally explore the prevalence rates, socioeconomic impacts and environmental links associated with paediatric airways diseases in the first 3 years of life.

## 2 Materials and Methods

A longitudinal prospective study was carried out. We enrolled 241 consecutive newborns whose mothers were admitted for delivery at ASST Santi Paolo and Carlo Hospital, Milan, Italy, from February to December 2016.

We included newborns of both sexes born after the 34th pregnancy week, with both Italian parents. Written informed consent was obtained by both parents of newborns.

Structured questionnaires were administered face to face to newborns' parents at birth, 1 year and at 3 years of life. At birth, we collected socio‐economic factors, anamnestic data of parents, pregnancy exposures and delivery characteristics, and children's characteristics at birth (see Table 1). At 1 and 3 years of age, we collected data on siblings, breastfeeding and solid foods, children's exposure to passive smoking and daycare attendance. In addition, children's history of AOM, URTI, bronchitis and bronchial asthma/wheezing during, respectively, the first and second/third years of life was reported by their parents.

**TABLE 1:** Characteristics of 241 newborns included in the study and their parents.

	*N* ^a^ (%)
Male sex	122 (50.6)
Gestational age (week), mean (SD)	39.2 (1.3)
Birth weight (g), mean (SD)	3266 (413)
Choanal exploration with nasogastric tube	84 (35.1)
Siblings	118 (53.4)
Day‐care attendance in the first year of life	78 (35.1)
Exclusive breastfeeding in the first year of life^b^	136 (61.3)
Breastfeeding with or without infant formula in the first year of life^b^	161 (72.5)
Breastfeeding at 1 year^c^	79 (35.8)
Maternal age at birth (yr), mean (SD)	33.7 (5.0)
Paternal age at birth (yr), mean (SD)	36.5 (5.7)
University maternal education	103 (42.9)
University paternal education	82 (34.3)
Mother passive smoking during pregnancy	39 (16.3)
Any pregnancy complication gestational diabetes	11(4.6)
Hypertension	9 (3.6)
Urinary/gynecological infections	39 (16.2)
Herpes symplex	10 (4.2)
Complications during delivery	29 (12.1)
Delivery	
Vaginal	186 (77.1)
Caesarean section	55 (22.9)
Elective	37 (67.9)
Emergency	18 (32.1)
Induction	57 (23.7)

^a^ Numbers in the Table are frequencies (percentages), unless otherwise specified. The sum may not add up to the total because of missing values. Percentages are calculated over the number of subjects with non‐missing information.

^b^ During the first year of life.

^c^ Until solid food introduction.

As part of the disease criteria, AOM was included among the conditions of interest, given its high incidence in paediatric age. While it is recognised that AOM has a distinct anatomo‐physiological trajectory compared to other airway diseases – being strongly linked to Eustachian tube immaturity in early life and often self‐resolving with age – we considered its early onset, frequency and possible recurrence to be clinically relevant. Its inclusion allows us to evaluate environmental and perinatal influences on the timing and burden of AOM, which may vary across individuals and could be informative for ENT clinical practice. Unlike asthma or allergy, whose chronic or immunological nature implies persistent predisposition, AOM will be analysed and interpreted separately, accounting for its distinct pathophysiology and natural resolution as the Eustachian tube matures.

Since there is no common validated questionnaire, paediatricians built an Italian‐written questionnaire including questions taken from the ISAAC study for asthma and allergies, and the Acute Respiratory Tract Infection Questionnaire for URTI, AOM and bronchitis [7, 8]. Parents were asked to bring all documentation relating to primary care visits, hospital stays and specialists' visits at the 1‐ and 3‐year evaluation.

At 1‐year follow‐up, allergy is intended only for food. A skin prick test for frequent allergens was performed during the last sampling (3y) in order to objectify allergic sensitisation.

The study was conducted in accordance with the Declaration of Helsinki and approved by the Institutional Review Board of ASST Santi Paolo and Carlo Hospital, Milan, Italy, number approval: 473/2016. Authors followed Wiley's Clinical Otolaryngology guidelines to write this paper.

### 2.1 Statistical Analysis

Descriptive statistics were used to describe data on newborns and their parents.

To maintain the correct temporal relationship between the exposures and the outcomes, we related smoking exposure in utero and in the first year of life, and kindergarten and breastfeeding in the first year of life with paediatric airways diseases occurrence during the second and/or third years of life. Sex‐adjusted log‐binomial regression models were used to derive the relative risks (RR) with their corresponding 95% confidence intervals (CI).

In case of cross‐sectional associations (e.g., breastfeeding and kindergarten in the first year of life and development of paediatric airways diseases during the first year of life), sex‐adjusted logistic regression models were used to estimate the odds ratios (OR) with their corresponding 95% CI.

To account for the effects of multiple testing, in further analyses the Benjamini–Hochberg procedure was used to control for the false discovery rate (FDR) at the desired level of 0.05.

All the analyses were performed using the SAS software, version 9.4 (SAS Institute Inc., Cary, NC, USA).

## 3 Results

Two hundred sevenpatients completed the three‐year follow‐up. Newborns and parents' characteristics as well as pregnancy and delivery characteristics are shown in Table 1.

Results about the association between the exposure to prenatal/perinatal and postnatal factors and the onset of URTI, AOM, asthma/wheezing, bronchitis and asthma at 1 and 3 years of life are resumed respectively in Tables 2 and 3.

**TABLE 2:** Association between delivery characteristics, siblings and breastfeeding and development of acute otitis media (AOM), upper respiratory tract infections (URTI), bronchitis, allergy and bronchial asthma/wheezing during the first year of life.

	AOM [*n* = 32]		URTI [*n* = 196]		Bronchitis [*n* = 58]	
	Cases/tot (%)	RR^a^ (95% CI)	Cases/tot (%)	RR^a^ (95% CI)	Cases/tot (%)	RR^a^ (95% CI)
Smoking exposure in utero and in the first year of life^b^						
No	25/150 (16.7)	1	137/150 (91.3)	1	42/150 (28.0)	1
Yes	7/70 (10.0)	0.55^c^ (0.23–1.35)	65/70 (92.9)	1.03^c^ (0.42–3.61)	19/70 (27.1)	0.96^c^ (0.51–1.81)
Delivery						
Vaginal	23/170 (13.5)	1	156/170 (8.2)	1	49/170 (28.8)	1
Caesarean section	9/51 (17.6)	1.34 (0.67–2.71)	47/51 (92.2)	1.00 (0.92–1.10)	13/51 (25.5)	0.89 (0.52–1.50)
Induction						
No	28/170 (16.5)	1	155/170 (91.2)	1	49/170 (28.8)	1
Yes	4/52 (7.7)	0.47 (0.17–1.27)	49/52 (94.2)	1.07 (0.95–1.20)	13/52 (25.0)	0.88 (0.52–1.50)
Complications during delivery						
No	29/194 (15.0)	1	179/194 (92.3)	1	58/194 (29.9)	1
Yes	3/27 (11.1)	0.83 (0.37–2.56)	24/27 (88.9)	0.96 (0.83–1.10)	4/27 (14.8)	0.47 (0.18–1.19)
Siblings						
No	15/103 (14.6)	1	93/103 (90.3)	1	18/103 (17.5)	1
Yes	17/118 (14.4)	0.98 (0.52–1.86)	110/118 (93.2)	1.04 (0.96–1.13)	44/118 (37.3)	2.13 (1.32–3.44)
Daycare attendance in the first year of life^c^						
No	16/128 (11.1)	1	129/144 (89.6)	1	33/144 (22.9)	1
Yes	16/78 (20.5)	2.20^d^ (1.02–4.73)	75/78 (96.2)	2.87^d^ (0.80–10.25)	29/78 (37.2)	1.96^d^ (1.07–3.58)
Exclusive breastfeeding in the first year of life^e^						
No	15/86 (17.4)	1	82/86 (95.4)	1	21/86 (24.4)	1
Yes	17/136 (12.5)	0.65^d^ (0.30–1.39)	122/136 (89.7)	0.43^d^ (0.14–1.35)	41/95 (30.2)	1.36^d^ (0.73–2.52)
Breastfeeding with or without infant formula in the first year of life^e^						
No	12/61 (19.7)	1	59/61 (96.7)	1	17/61 (27.9)	1
Yes	20/161 (12.4)	0.55^d^ (0.25–1.21)	145/161 (90.1)	0.31^d^ (0.07–1.40)	45/161 (28.0)	1.03^d^ (0.53–1.99)
Breastfeeding at 1‐year						
No	24/142 (16.9)	1	136/142	1	46/142 (32.4)	1
Yes	8/79 (10.1)	0.55^d^ (0.23–1.30)	67/79	0.25^d^ (0.09–0.69)	16/79 (20.3)	0.53^d^ (0.28–1.02)

	Allergy to food (*n* = 14)		Bronchial asthma/wheezing (*n* = 23)	
	Cases/tot (%)	RR^a^ (95% CI)	Cases/tot (%)	RR^a^ (95% CI)
Smoking exposure^b^				
No	10/149 (6.7)	1	22 (14.7)	1
Yes	4/70 (5.7)	0.85 (0.28–2.62)	3 (4.3)	0.29 (0.09–0.94)
Delivery				
Vaginal	10/170 (5.9)	1	21/170 (12.4)	1
Caesarean section	4/50 (8.0)	1.36 (0.44–4.15)	5/51 (9.8)	0.81 (0.32–2.04)
Induction				
No	9/169 (5.3)	1	23/170 (13.5)	1
Yes	5/52 (9.6)	1.80 (0.63–5.2)	3/52 (5.8)	0.43 (0.13–1.36)
Complications during delivery				
No	14/194 (7.2)		25/194 (12.9)	1
Yes	0/26 (0.0)	ne	1/727 (3.7)	0.33 (0.04–2.38)
Siblings				
No	4/102 (3.9)	1	5/103 (4.9)	1
Yes	10/118 (8.5)	2.17^d^ (0.70–6.73)	21/118 (17.8)	3.58^d^ (1.41–9.14)
Daycare attendance in the first year of life^c^				
No	5/144 (3.5)	1	16/144	1
Yes	9/77 (11.7)	3.70^d^ (1.19–11.5)	10/78	1.25 (0.53–2.93)
Exclusive breastfeeding in the first year of life^e^				
No	5/86 (5.8)	1	5/86 (5.8)	1
Yes	9/135 (6.7)	1.16^d^ (0.33–2.91)	21/136 (15.4)	2.89^d^ (1.04–7.98)
Breastfeeding with or without infant formula in the first year of life^e^				
No (only infant formula)	5/61 (8.2)	1	4/61 (6.6)	1
Yes	9/160 (5.6)	0.67^d^ (0.21–2.08)	22 (13.7)	2.16^d^ (0.71–6.57)
Breastfeeding at 1‐year				
No	11/142 (7.8)	1	17/142 (12.0)	1
Yes	3/78 (3.9)	0.48^d^ (0.13–1.76)	9/79 (11.4)	0.94^d^ (0.40–2.23)

Abbreviations: CI: confidence interval; ne: not estimable; RR: relative risk.

^a^ Adjusted for sex.

^b^ Maternal smoking during pregnancy, or maternal passive smoking exposure during pregnancy, or children's passive smoking exposure during the first year of life.

^c^ Sex‐adjusted odds ratio from a logistic regression model to account for the cross‐sectional association.

^d^ Odds ratio from logistic regression model, adjusted for sex.

^e^ Until solid food introduction.

**TABLE 3:** Relative risk (RR) and corresponding 95% confidence intervals (CI) for the association between delivery characteristics, siblings and breastfeeding and development of acute otitis media (AOM), upper respiratory tract infections (URTI), bronchitis and bronchial asthma/wheezing during the first 3 years of life^a^ and positivity to the skin prick test at the 3 year follow‐up visit.

	AOM [*n* = 89]		URTI [*n* = 209]		Bronchitis [*n* = 99]	
	Cases/tot (%)	RR^b^ (95% CI)	Cases/tot (%)	RR^b^ (95% CI)	Cases/tot (%)	RR^b^ (95% CI)
Smoking exposure^a^, ^c^						
No	50/138 (36.2)	1	51/137 (37.2)	1	46/138 (33.3)	1
Yes	24/63 (38.1)	1.05 (0.71–1.54)	37/63 (58.7)	1.59 (1.18–2.14)	23/63 (36.5)	1.10 (0.73–1.64)
Delivery						
Vaginal	68/158 (43.0)	1	159/169 (94.0)	1	76/161 (47.2)	1
Caesarean section	21/48 (43.8)	1.01 (0.70–1.46)	49/51 (96.1)	1.03 (0.93–1.15)	23/46 (50.0)	1.06 (0.76–1.48)
Induction						
No	73/160 (45.6)	1	159/169 (94.1)	1	76/160 (47.5)	1
Yes	16/47 (34.0)	0.74 (0.48–1.15)	50/52 (96.2)	1.03 (0.93–1.15)	23/48 (47.9)	1.02 (0.73–1.43)
Complications during delivery						
No	74/179 (41.3)		183/193 (94.8)	1	87/181 (48.1)	1
Yes	15/27 (55.6)	1.35 (0.93–1.98)	25/27 (92.6)	0.97 (0.87–1.07)	12/26 (46.2)	0.95 (0.61–1.48)
Siblings						
No	39/95 (41.1)		96/102 (94.1)	1	42/95 (44.2)	1
Yes	49/110 (44.5)	1.08 (0.78–1.48)	112/118 (94.9)	1.02 (0.96–1.08)	56/111 (50.5)	1.13 (0.85–1.52)
Daycare attendance in the first year of life^a^, ^d^						
No	40/130 (30.8)	1	52/130 (40.0)	1	47/130 (36.2)	1
Yes	34/73 (46.6)	1.51 (1.06–2.16)	38/72 (52.8)	1.29 (0.96–1.75)	22/73 (30.1)	0.84 (0.55–1.28)
Exclusive breastfeeding in the first year of life^a^, ^e^						
No	29/76 (38.2)	1	36/76 (47.4)	1	22/76 (29.0)	1
Yes	45/127 (35.4)	0.93 (0.64–1.36)	54/126 (42.9)	0.92 (0.68–1.26)	47/127 (37.0)	1.27 (0.84–1.94)
Breastfeeding with or without infant formula in the first year of life^a^, ^e^						
No (only infant formula)	20/57 (35.1)	1	28/57 (49.1)	1	14/57 (24.6)	1
Yes	54/146 (37.0)	1.06 (0.70–1.61)	62/145 (42.8)	0.90 (0.65–1.24)	55/146 (37.7)	1.53 (0.93–2.53)
Breastfeeding at 1‐year^a^						
No	45/127 (33.4)	1	57/126 (45.2)	1	40/127 (31.5)	1
Yes	29/76 (38.2)	1.08 (0.74–1.56)	33/76 (43.4)	0.97 (0.71–1.33)	29/76 (38.2)	1.21 (0.83–1.78)

	Bronchial asthma/wheezing [*n* = 43]		Positive skin prick test [*n* = 17]	
	Cases/tot (%)	RR^b^ (95% CI)	Cases/tot (%)	RR^b^ (95% CI)
Smoking exposure in utero and in the first year of life^a^, ^c^				
No	20/138 (14.5)	1	12/118 (10.2)	1
Yes	4/63 (6.4)	0.44 (0.16–1.23)	5/36 (13.9)	1.36 (0.51–3.60)
Delivery				
Vaginal	34/157 (21.7)	1	12/121 (9.9)	1
Caesarean section	9/46 (19.6)	0.92 (0.48–1.76)	5/35 (14.3)	1.43 (0.54–3.80)
Induction				
No	36/158 (22.8)	1	15/121 (12.4)	1
Yes	7/46 (15.2)	0.65 (0.31–1.36)	2/36 (5.6)	0.45 (0.10–1.87)
Complications during delivery				
No	39/178 (21.9)	1	16/135 (11.9)	1
Yes	4/25 (16.0)	0.78 (0.30–2.00)	1/21 (4.8)	0.41 (0.06–2.91)
Siblings				
No	13/93 (14.0)	1	8/74 (10.8)	1
Yes	30/110 (27.3)	1.96 (1.09–3.53)	9/81 (11.1)	1.04 (0.42–2.55)
Daycare attendance in the first year of life^a^, ^d^				
No	31/130 (23.9)	1	14/102 (13.7)	1
Yes	12/74 (16.2)	0.72 (0.39–1.32)	3/54 (5.6)	0.41 (0.12–1.36)
Exclusive breastfeeding in the first year of life^a^, ^e^				
No	9/76 (11.8)	1	6/63 (9.5)	1
Yes	15/127 (11.8)	1.00 (0.46–2.18)	11/93 (11.8)	1.24 (0.48–3.17)
Breastfeeding with or without infant formula in the first year of life^a^, ^e^				
No (only infant formula)	7/57 (12.3)	1	4/45 (8.9)	1
Yes	17/146 (11.6)	0.95 (0.42–2.18)	13/111 (11.7)	1.31 (0.45–3.80)
Breastfeeding at 1‐year^a^				
No	14/127 (11.0)	1	11/99 (11.1)	1
Yes	10/76 (13.2)	1.19 (0.56–2.55)	6/57 (10.5)	0.95 (0.37–2.42)

Abbreviations: CI: confidence interval; RR: relative risk.

^a^ For the variables smoking exposure, daycare and breastfeeding, we evaluated the association with the development of the outcomes within the first and third year of life (AOM: 75; URTI: 90, bronchitis: 70, asthma: 24).

^b^ Adjusted for sex.

^c^ Maternal smoking during pregnancy, or maternal passive smoking exposure during pregnancy, or children's passive smoking exposure during the first year of life.

^d^ During the first year of life.

^e^ Until solid food introduction.

### 3.1 Influencing Factors and Development of Diseases During the First Year

**FIGURE 1:**
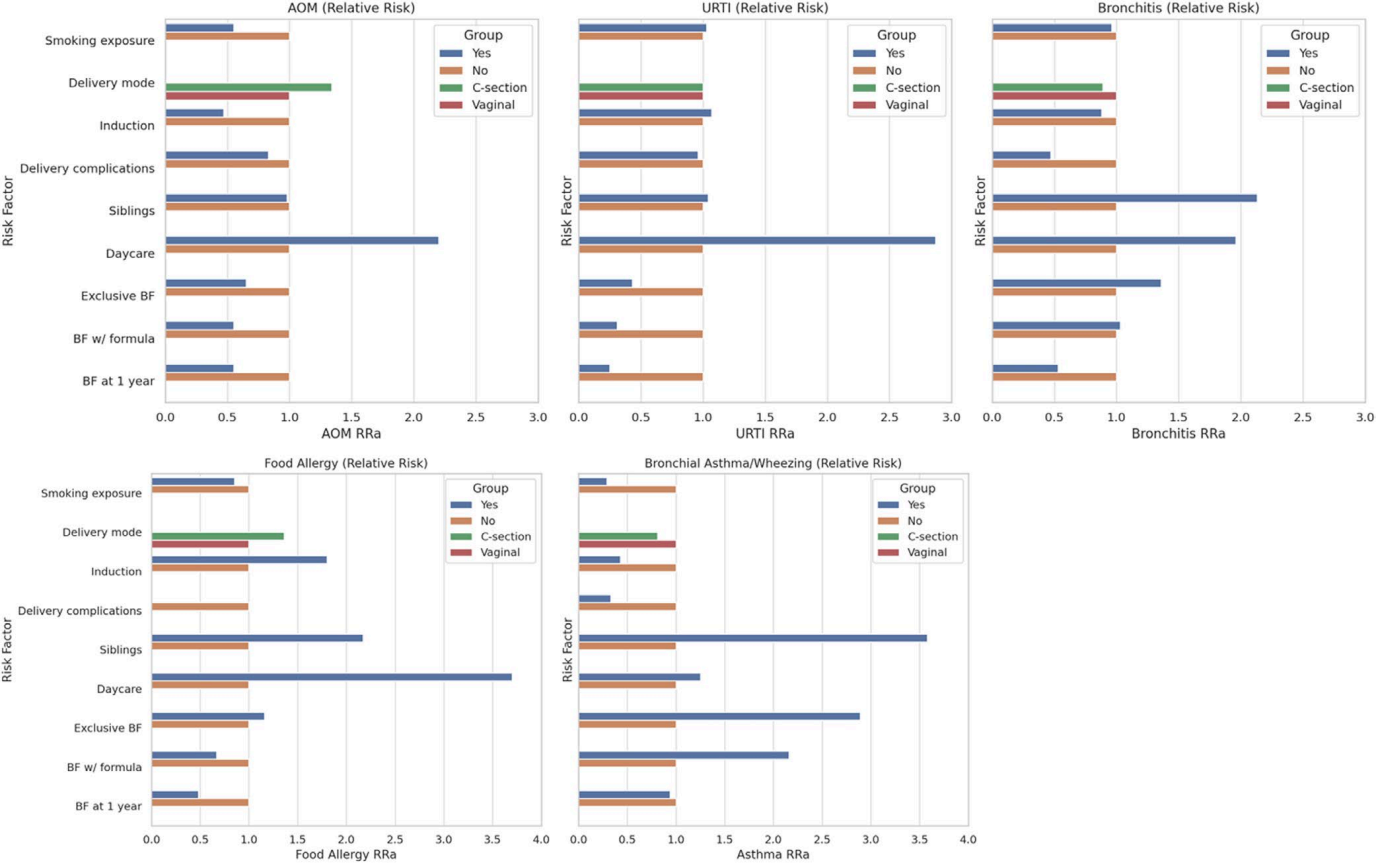
Graphic representation of the association (RR) between risk factors and diseases evaluated at 1 year of life.

Having siblings was associated with an increased risk of bronchitis (RR 2.13, 95% CI: 1.32–3.44) and asthma/wheezing (RR 3.58, 95% CI: 1.41–9.14) during the first year of life (Figure 1). Asthma was also directly associated with exclusive breastfeeding (OR 2.89, 95% CI: 1.04–7.98). Smoking exposure (intended as maternal smoking during pregnancy, or maternal passive smoking exposure during pregnancy, or children passive smoking exposure during the first year of life) was inversely associated with the odds of asthma/wheezing (OR 0.26, 95% CI: 0.07–0.90), attending kindergarten was directly associated with the odds of allergy (OR 3.7, 95% CI: 1.19–11.5), and breastfeeding at 1 year was inversely associated with the odds of URTI (OR 0.25, 95% CI: 0.09–0.69). No other significant association was detected (Figure 1).

### 3.2 Influencing Factors and Development of Diseases at 3 Years of Life

**FIGURE 2:**
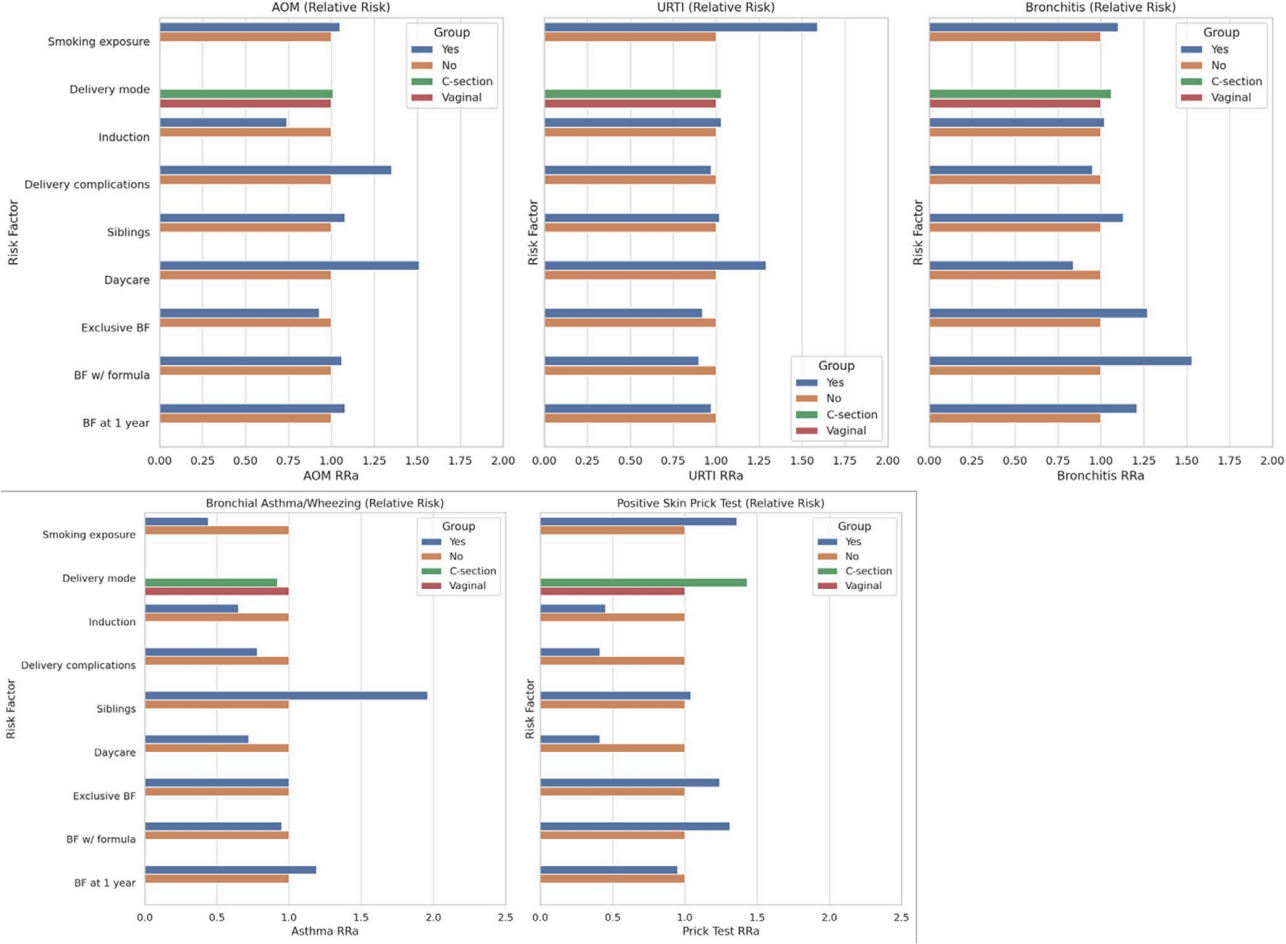
Graphic representation of the association (RR) between risk factors and diseases evaluated at 3 years of life.

Kindergarten attendance was associated with a non‐significantly lower risk of positive skin prick test to inhalants (RR 0.41, 95% CI: 0.12–1.36) and with an increased risk of AOM (RR 1.51, 95% CI: 1.06–2.16) (Figure 2). Child exposure to smoking in utero and during the first year of life increased the risk of URTI up to 3 years (RR 1.59, 95% CI: 1.18–2.14), and having siblings increased the risk of asthma/wheezing (RR 1.96, 95% CI: 1.09–3.53). No association was found with feeding practices and the other factors analysed.

In sensitivity analyses, no significant association remained after controlling for the FDR. FDR‐adjusted *p*‐values < 0.10 but higher than 0.05 were found for the RR of the association between siblings and bronchitis during the first year and between smoking exposure in utero and in the first year of life and URTI during the second and/or third years of life.

## 4 Discussion

The high incidence of paediatric diseases is a growing public health concern with wide‐ranging impact on children's well‐being, but to our knowledge, no authors focused on multiple factors applied to multiple pathologies which may impact on the ENT daily practice, especially with prospective longitudinal studies [4].

The role of early‐life exposures in shaping immune responses and susceptibility to airway infections is well documented. Recently, nutritional status and dietary interventions have been increasingly studied as modifiable factors influencing the burden and duration of respiratory tract infections. A recent EAACI taskforce systematic review highlighted that although evidence remains limited, specific nutrients such as zinc may offer modest benefits in selected populations, underscoring the need for targeted prevention strategies from early childhood.

### 4.1 Smoking Exposure

Smoke crosses the placenta and reaches amniotic fluid and the respiratory tract: fetal serum nicotine concentrations can be higher by 15% than in maternal serum [9]. Our data show a higher risk of URTIs during the first 3 years of life in children exposed to smoke (Table 3, Figure 2), confirming already available evidence of smoking exposure being a significant risk factor for paediatric respiratory diseases such as URTIs and in particular rhinitis [10].

Numerous studies reported a strong positive association between exposure to smoke during pregnancy and the development of paediatric asthma, as for Moore [6]. A recent review found 4 articles all reporting a positive link between prenatal environmental smoking exposure and an increased risk of asthma in Chinese children, but none of them discussed the age of onset [11]. Surprisingly, our analysis showed a protective role of smoking exposure for asthma at 1 year (RR 0.29, Table 2). This mismatch may be due to the fact that in the literature there is no clarification about the age of onset of asthma for those patients; our evidence may have been taken too prematurely and therefore missing a later development of the disease.

AOM among children is attributable to passive smoking exposure by some authors [12]. Instead, a recent study among 7863 children did not evidence any link between prenatal smoking and the risk of AOM, but instead a correlation between AOM and postnatal household smoking [13]. We did not find any correlation between prenatal smoking exposure and the onset of AOM (Figure 1).

Among the diseases analysed, AOM occupies a unique place, both anatomically and temporally, in paediatric airway pathology. Unlike chronic conditions such as asthma or allergic rhinitis – which are influenced by long‐term inflammatory mechanisms and immunological predisposition – AOM is often the result of functional and mechanical immaturity of the Eustachian tube in early childhood. The tube's horizontal orientation, reduced length and more compliant structure in infants predispose to fluid retention and secondary infections of the middle ear.

We therefore included AOM in our analysis to map its occurrence and association with modifiable risk factors – such as daycare attendance, passive smoke exposure and feeding patterns – during the critical window of early childhood. However, its interpretation must differ from that of asthma or allergic diseases. While the latter may reflect an evolving global airway inflammatory process, AOM should be understood within its distinct developmental timeline, and its presence in our dataset is intended to enrich our understanding of ENT‐specific risks and exposures during the maturation of the paediatric airway.

Our analysis also did not find a link with the development of allergies to food nor to inhalants (Figures 1 and 2). Accordingly, a systematic review by Lau et al. evidenced that parental smoking was generally not associated with the risk of food allergy by age 3 years [14].

Poor literature is provided among the bronchitis–smoking exposure association. A paper highlighted the exposure to second‐hand smoke as a potential cause of bronchitis and pneumonia [15]. We could not assess a correlation between smoking and bronchitis in the first 3 years of life.

### 4.2 Siblings

A recent systemic review highlighted that being second‐born or later is associated with an increased risk of temporary wheezing in infancy. In contrast, having ≥ 1 older sibling was marginally protective for subjects aged ≥ 6 years in terms of asthma, although not statistically significant [16]. These associations appear to have weakened in the last 20 years compared to the evidence before, possibly due to lifestyle changes and socioeconomic development.

Interestingly, we found an association between having siblings and a higher risk of asthma and wheezing at both 1 and 3 years (Tables 2 and 3). However, we could not show a long‐term role in asthma maintenance because of the limited follow‐up.

Another piece of evidence associated with siblings is a higher risk of bronchitis during the first year of life; this outcome does not seem to be relevant yet at 3 years (Tables 2 and 3). Accordingly, a consensus conference of 2010 among paediatricians states that having siblings is a risk factor for bronchiolitis, without any specification about age [17].

The 2023 systematic review also highlighted how siblings are protective against developing allergies [18]; we did not find this kind of correlation in our analysis, not at 1 nor at 3 years.

Very little is reported in the literature about the risk of developing AOM among siblings: only a study of 7863 children reported that the presence of siblings increased the odds of AOM [13]. Our analysis did not find any correlation in these terms.

### 4.3 Breastfeeding

Breastfeeding is another factor that has been extensively studied for its potential protective effects against paediatric diseases.

A systematic review evidenced that both exclusive and nonexclusive prolonged breastfeeding (6 months or more) decreased the risk of rhinitis. The long‐term (12 months or more) nonexclusive breastfeeding lowers the risk of rhinitis compared to 12 months or fewer, while long‐term exclusive breastfeeding did not show the same protective effect [19].

Accordingly, in our analysis we found that both exclusive and non‐exclusive breastfeeding and also breastfeeding maintained at 1 year play a protective role against URTI at 1 year, but statistically significant only for breastfeeding prolonged until 1 year (Table 2). At 3 years' follow‐up outcomes are not significant anymore (Table 3).

About asthma, our data revealed that exclusive breastfeeding is associated with a higher rate of asthma and wheezing at 1 year (RR 2.89, see Table 2), and loses its relation at 3 years.

Literature is controversial. Many studies attribute to breastfeeding a protective role against asthma. Also, the timing of solid food introduction is important: the sooner, the higher is the risk of suffering from wheezing [20]. On the other hand, some research offers opposite findings [21]. These divergent outcomes may be attributed to variations in the size of the sample, the focus of determinants, participant age and confounding factors. For instance, a clinical trial found that children who were exclusively breastfed and overweight at the time of the study were more prone to developing asthma [22].

Breastfeeding also showed to decrease AOM odds, most notably in the first 2 months of life [13]. In our analysis, since we did not introduce a follow‐up period of months, we could not assess this correlation. Instead, we could not show any kind of association with AOM, allergy, or bronchitis at 1 and 3 years of life (Tables 2 and 3).

Continuing to promote breastfeeding and its benefits may contribute to a decrease in the prevalence of infections, even though we found a positive association with asthma in the first year of life.

### 4.4 Daycare Attendance

Attending daycare centers has been linked to an increased risk of URTIs and other infectious diseases in children: the exposure to a larger number of peers and their associated germs can contribute to a higher incidence of these diseases. Anyway, an interesting prospective population‐based study on 1946 children found no association between daycare attendance and childhood asthma [23]. Our analysis agrees with these findings in the 3‐year follow‐up (Tables 2 and 3, Figure 2). Probably, as previously debated for breastfeeding, daycare attendance cannot be considered as an independent risk factor for developing asthma; it is likely that the combination with other influencers may drive toward future asthma/wheezing.

When exploring the link of daycare attendance with atopy, very few old studies are available. A review concluded that the relation is unclear [24]. Interestingly, in our analysis we found a protective role of attending daycare in developing allergies to inhalants and food at 3 years of life (Table 3, Figure 2), while we evidenced a higher risk of food allergy at 1 year (Table 2). Nevertheless, it has to be clarified that the skin prick test may encounter a certain rate of false positives and negatives, with an estimated sensitivity ranging from 60% to 79% and specificity ranging from 68% to 69%, altering the strength of evidence [25].

Our study also showed a higher risk for children attending daycare in developing AOM at 3 years (RR 1.51, Table 3, Figure 2). Literature reinforces our evidence [26], showing how the exposure to germs and pollutants clearly raises the risk of infections for infants. Accordingly, we found a higher risk for these patients in getting bronchitis (RR 1.96, Table 1, Figure 1), as shown in literature [27], while at 3 years the risk disappears, maybe due to the maturation of the immune system, as hypothesised by some authors (Figure 2).

To summarise, attendance to daycare seems to expose children to higher rates of upper and lower infections, while it appears to protect against the development of allergies to inhalants at 3 years of life, maybe enhancing a Th1 response rather than a Th2 one.

Among the study limitations, given the number of statistical analyses conducted, some chance findings are expected. In sensitivity analyses, no significant association remained after controlling for the FDR. Thus, caution is needed in interpreting our positive findings.

Despite the clinical relevance of early‐life respiratory diseases, evidence guiding effective preventive strategies remains limited. Most available studies focus on single conditions, often with retrospective designs or limited follow‐up. As recently pointed out by an EAACI taskforce, even systematic reviews addressing acute respiratory infections in children reveal a general scarcity of high‐quality, consistent data across populations and settings [28]. This highlights the need for prospective, multi‐dimensional studies–such as the present one–to better characterise risk patterns in the first years of life.

## 5 Conclusions

The high incidence of inflammatory and allergic paediatric diseases underscores the importance of identifying predictive factors to mitigate their impact.

Our analysis found a conflicting role of smoking exposure and breastfeeding, the former protective for asthma and risk for URTIs, the latter the opposite. Having siblings appeared to raise the risk of having asthma and bronchitis; attendance to daycare seems to expose children to a higher rate of allergies to food at 1 year and of upper and lower infections, while it is protective against allergies to inhalants at 3 years.

The present findings support a growing call to action in paediatric care to target modifiable risk factors and adopt a preventive, trait‐based model already in early childhood. This is consistent with the EUFOREA consensus, which outlines remission and disease modification as new, feasible goals in asthma clinical practice [2]. Further research is needed to refine our understanding of these factors and their interplay in the context of paediatric diseases.

## Author Contributions

Every author gave (a) substantial contributions to the conception or design of the work; or the acquisition, analysis, or interpretation of data for the work; (b) drafting the work or revising it critically for important intellectual content; (c) final approval of the version to be published; and (d) Agreement to be accountable for all aspects of the work in ensuring that questions related to the accuracy or integrity of any part of the work are appropriately investigated and resolved.

## Ethics Statement

This paper follows the core practices of the Committee on Publication Ethics (COPE).

## Consent

Written informed consent was obtain by both parents of newborns.

## Conflicts of Interest

The authors declare no conflicts of interest.

## Data Availability

The data that support the findings of this study are available from the corresponding author upon reasonable request.
